# P-1645. Appropriateness of Ambulatory Antibiotic Prescriptions Given Outside of Formal Patient-Clinician Encounters

**DOI:** 10.1093/ofid/ofae631.1811

**Published:** 2025-01-29

**Authors:** Nathan R Shively, Dustin R Carr, Matthew A Moffa, G E O R G E BCHECH, Ryan Rothman, Edmund Appiah-Kubi, Sophia Barber, Vivian Kalu, James J Nagel, Derek N Bremmer, Carley Buchanan, Tamara Trienski, Shenjun Zhu, Thomas L Walsh

**Affiliations:** Allegheny Health Network, Pittsburgh, Pennsylvania; Allegheny General Hospital, Pittsburgh, Pennsylvania; Allegheny Health Network, Pittsburgh, Pennsylvania; Allegheny General Hospital/Allegheny Health Network, PITTSBURGH, Pennsylvania; Allegheny General Hospital/Allegheny Health Network, PITTSBURGH, Pennsylvania; Allegheny health network, Pittsburgh, Pennsylvania; Allegheny Health Network, Pittsburgh, Pennsylvania; Allegheny Health Network, Pittsburgh, Pennsylvania; Allegheny General Hospital, Pittsburgh, Pennsylvania; Allegheny General Hospital, Pittsburgh, Pennsylvania; Allegheny Health Network, Pittsburgh, Pennsylvania; Allegheny General Hospital, Pittsburgh, Pennsylvania; Allegheny Health Network, Pittsburgh, Pennsylvania; Allegheny Health Network, Pittsburgh, Pennsylvania

## Abstract

**Background:**

There are limited published data on the frequency and appropriateness of outpatient antibiotic prescriptions ordered outside of formal patient-clinician encounters.

**Figure:**
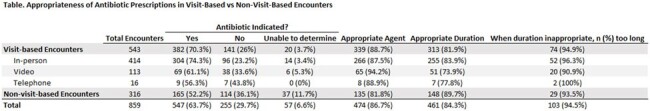

**Methods:**

A random sample of oral antibiotics prescribed to adult patients from 4 primary care practices were manually reviewed for appropriateness. The need for any antibiotic, as well as the appropriateness of agent selection and duration, were compared between visit-based (formal in-person, video, or telephone encounters) and non-visit-based encounters.

**Results:**

Of the 859 prescriptions reviewed, 543 (63.2%) were generated from visit-based encounters (48.2%, 13.2% and 1.9% were from in-person, video, and telephone visits, respectively) and 316 (36.8%) were non-visit-based. Overall, 29.7% of prescriptions were written despite no antibiotic indication (Table). Antibiotics were indicated in 382/543 (70.3%) of visit-based encounters, vs 165/316 (52.2%) of non-visit-based encounters (P< 0.0001). When an antibiotic was indicated, agent selection was more frequently appropriate in visit-based encounters (88.7% vs 81.8%, P=0.006), with no difference in appropriate duration (81.9% vs 89.7%, P=0.49). When duration was inappropriate, it was too long in 94.5% of cases. Among visit-based prescribing, antibiotic prescriptions were indicated more frequently from in-person encounters as compared to virtual (combined video and telephone) encounters (73.4% vs 60.5%, P=0.008). Compared to visit-based antibiotic prescriptions, significantly fewer non-visit-based antibiotic prescriptions were associated with any diagnosis code (543/543 (100%) vs 189/316 (59.8%), P< 0.0001). Azithromycin and fluoroquinolones were more likely to be inappropriately chosen agents compared to other antibiotics (46.7% vs 12.4%, P< 0.0001, and 45.5% vs 12.4%, P< 0.0001, respectively).

**Conclusion:**

Compared to antibiotic prescriptions from formal patient-clinician encounters, antibiotic prescriptions generated from non-visit-based encounters were significantly less indicated, with less appropriate agent selection, and were frequently not associated with a diagnosis code. Outpatient stewardship efforts should target non-visit-based encounters.

**Disclosures:**

**Dustin R. Carr, PharmD, BCPS, BCIDP**, Merck: Advisor/Consultant **Derek N. Bremmer, PharmD, BCIDP**, Thermo Fischer Scientific: Expert Testimony

